# Improving Intraoperative Compliance With Critical View of the Myopectineal Orifice Criteria Using the V–M Pathway During Laparoscopic Inguinal Hernia Repair

**DOI:** 10.3389/jaws.2026.16223

**Published:** 2026-03-25

**Authors:** Kryspin Mitura, Laura Kacprzak, Marta Wojcik, Lidia Mitura, Michal Romanczuk, Bernard Mitura, Orest Lerchuk, Volodymyr Khomyak, Orest Chemerys, Piotr Niecikowski

**Affiliations:** 1 University in Siedlce, Siedlce, Poland; 2 Hospital Franciszek Raszeja, Department of General Surgery, Poznan, Poland; 3 Uniwersytet Medyczny w Lublinie, Lublin, Poland; 4 Jagiellonian University Collegium Medicum, Cracow, Poland; 5 L’vivs’kij Nacional’nij Medicnij Universitet Imeni Danila Galic’kogo, Lviv, Ukraine

**Keywords:** critical view of myopectineal orifice, CVMPO criteria, intraoperative decision-making, laparoscopic inguinal hernia repair, spatial mnemonic

## Abstract

**Background:**

The CVMPO criteria were developed to standardize critical quality steps during laparoscopic inguinal hernia repair. However, consistent intraoperative compliance remains challenging, particularly during prolonged or technically difficult procedures, or under cognitive fatigue. Checklist-based approaches may be difficult to recall and apply in real time.

**Methods:**

A prospective observational study included 211 consecutive laparoscopic inguinal hernia repairs performed using a standardized technique. An original spatial cognitive framework, the V–M Pathway, mapping CVMPO criteria onto the laparoscopic view of the inguinal region, was applied as a deliberate intraoperative pause before mesh placement. Time required to verbally recall all nine CVMPO criteria was measured intraoperatively. All procedures were routinely recorded in full from the beginning to the end of the operation. Surgeon-identified corrective actions prompted by CVMPO recall were documented during the procedure. The time required to implement corrective actions was determined postoperatively based on independent review of the complete operative video recordings. All procedures underwent *post hoc* video verification to confirm objective adherence to the CVMPO criteria.

**Results:**

Complete recall of all nine CVMPO criteria using the V–M Pathway was achieved in all procedures, with a median recall time of 58 s (IQR 52–64). Intraoperative recall prompted corrective actions in 20.4% of cases. Among procedures requiring corrective actions, the median time required to complete all corrections was 46 s (IQR 38–81). Independent video review confirmed complete adherence to all CVMPO criteria in 97.2% of procedures. Mesh placement was performed only after CVMPO confirmation in all cases.

**Conclusion:**

The V–M Pathway supported rapid intraoperative recall of CVMPO criteria, prompted timely corrective actions, and was associated with high video-verified adherence to predefined quality criteria. Importantly, the additional time required for recall and corrective actions represented only a small fraction of total operative time, suggesting that integration of the pathway is feasible without major workflow disruption. This simple spatial cognitive aid may support intraoperative verification of quality criteria during laparoscopic inguinal hernia repair without disrupting operative workflow.

## Introduction

Laparoscopic inguinal hernia repair has become a standard approach worldwide; however, ensuring consistent adherence to established quality criteria remains challenging [1, 2]. The Critical View of the Myopectineal Orifice (CVMPO) criteria were developed to standardize critical intraoperative steps and reduce technical variability, yet their complete and systematic application is not always achieved in routine clinical practice, particularly during prolonged procedures or along the learning curve [3, 4].

With the widespread adoption of the TAPP approach, which is increasingly becoming a standard technique for inguinal hernia repair, the need for effective tools that limit technical variability becomes particularly important [5]. Previous experience from open Lichtenstein repair has shown that, despite a well-defined operative concept, gradual individual modifications introduced by surgeons may lead to substantial heterogeneity in technique [6]. Failure to consistently fulfill predefined technical criteria may adversely affect clinical outcomes, particularly with regard to hernia recurrence and chronic postoperative pain [7]. As minimally invasive hernia surgery continues to expand, reliance on individual memory and interpretation alone may reintroduce avoidable imprecision, underscoring the need for standardized intraoperative cognitive support [8].

One important reason for incomplete adherence is cognitive load [9]. During minimally invasive surgery, surgeons must simultaneously process anatomical orientation, technical execution, and decision-making under conditions of fatigue, time pressure, variable anatomy, and objective technical difficulty [8]. In such settings, checklist-based criteria may be known conceptually but are difficult to recall and verify intraoperatively, especially toward the end of the procedure when attention naturally declines.

To address this gap between knowledge and behavior, we developed an original spatial cognitive framework, referred to as the V–M Pathway, that transforms the CVMPO checklist into an anatomically mapped intraoperative pathway. By aligning mnemonic elements with the laparoscopic view of the myopectineal orifice of Fruchaud, the V–M Pathway provides a continuous visual–spatial reference designed to support real-time recall and execution of all CVMPO criteria. The aim of this study was to evaluate intraoperative usability and workflow integration of the V–M Pathway during laparoscopic inguinal hernia repair.

## Materials and Methods

### Study Design

This prospective observational study included consecutive laparoscopic inguinal hernia repairs performed between March 2023 and September 2025 using a standardized operative transabdominal preperitoneal (TAPP) technique. The objective was to evaluate the intraoperative usability and behavioral impact of a spatial cognitive framework, the V–M Pathway, designed to support recall and execution of the CVMPO criteria.

All consecutive patients undergoing laparoscopic inguinal hernia repair during the study period were eligible for inclusion. Procedures in which the V–M Pathway could not be applied due to technical constraints or incomplete video recording were excluded from analysis. The number of excluded procedures and reasons for exclusion are reported in the Results section. Procedures were performed by multiple surgeons within the department, including both board-certified surgeons and residents performing procedures under supervision, reflecting routine clinical practice.

### Development of the V–M Pathway

An original spatial cognitive framework, referred to as the V–M Pathway, was developed to facilitate real-time intraoperative recall of the CVMPO criteria. The framework preserves the letters “V” and “M” from the CVMPO acronym and transforms them into a continuous spatial pathway. By inverting the letter “V” along its horizontal axis, a zigzag-shaped pathway with five angular deflections extending from the medial to the lateral aspect of the myopectineal orifice is created.

This pathway is mapped onto the laparoscopic view of the right inguinal region, extending from the pubic symphysis to the anterior superior iliac spine, with each segment corresponding to a specific anatomical area and CVMPO element. Rather than functioning as a checklist, the V–M Pathway serves as a visual–spatial cue designed to support recall and enable rapid, systematic verification of all CVMPO criteria at a defined intraoperative pause point. For procedures performed on the contralateral side, the V–M Pathway is applied as a mirror image of the described configuration.

In our institution, CVMPO principles constitute a routine component of TAPP training rather than a separately taught module; therefore, surgeons participating in the study were already familiar with the CVMPO criteria prior to study initiation. Introduction of the V–M Pathway did not require formal training sessions. Instead, surgeons were introduced to the spatial mapping concept during routine departmental meetings, after which the framework was incorporated into routine clinical practice.

The pathway functions as a spatial mnemonic rather than an external checklist; therefore, surgeons were expected to possess prior conceptual knowledge of CVMPO criteria. No written checklist was used intraoperatively, and verification relied on internalized knowledge supported by spatial orientation within the operative field. Surgeons typically reported becoming comfortable with the pathway after a small number of procedures, although formal learning-curve assessment was not performed.

### Intraoperative Application of the V–M Pathway

During each procedure, the operating surgeon completed hernia dissection and proceeded to the final inspection stage immediately before mesh placement. Standardized mesh dimensions of at least 10 × 15 cm were used in all procedures, ensuring consistent fulfillment of CVMPO coverage requirements. At this point, a deliberate intraoperative pause was performed.

**FIGURE 1:**
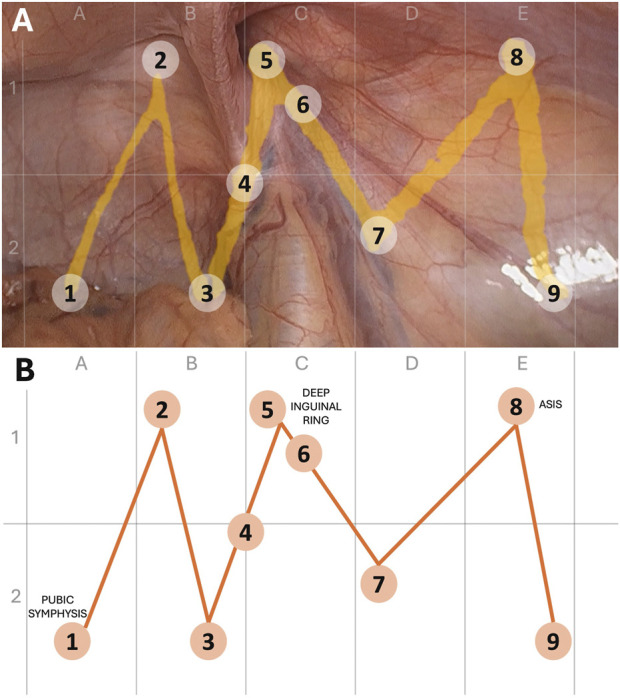
Sector-based representation of the V–M Pathway mapped onto the myopectineal orifice. **(A)** Laparoscopic view of the right myopectineal orifice during TAPP repair with pathway mapping. **(B)** Simplified schematic diagram illustrating sector division and pathway configuration. The operative field is divided into five vertical sectors (A–E) and two horizontal levels (upper [1] and lower [2]) to facilitate spatial orientation. Numbered points (1–9) correspond to individual CVMPO criteria arranged along the V–M Pathway. For procedures performed on the contralateral side, the pathway is applied as a mirror image.

For spatial orientation, the laparoscopic view of the operative field is divided into five vertical sectors (A–E). Sector C represents the central region; sectors A and B the medial region, and sectors D and E the lateral region. In addition, each sector is bisected by a horizontal line into an upper (ventral, 1) and lower (dorsal, 2) portion, further refining localization of specific CVMPO steps within the operative field (Figure 1).

Each point of the V–M Pathway is positioned to correspond to the anatomical location at which the relevant CVMPO-related action should be considered. For example, step 1 is located in sector A2, corresponding to the pubic symphysis and Cooper ligament, whereas step 5 is located in sector C1 and corresponds to the deep inguinal ring and indirect hernia sac dissection. Some pathway points serve a conceptual rather than strictly focal role. Step 7, located in sector D2, emphasizes the need to ensure adequate dissection of the inferolateral portion of the myopectineal orifice to allow proper mesh deployment across sectors D2, E1, and E2. Step 8 is positioned above the inter-ASIS line in sector E1, directly overlying the triangle of pain, and serves as a reminder to provide sufficient mesh coverage (including this point) and to avoid penetrating mesh fixation in the region beneath this point. Step 9 marks the terminal portion of the pathway at the anticipated line of peritoneal closure and prompts final verification of adequate mesh size.

During this pause, the operating surgeon verbally reviewed the CVMPO criteria using the V–M Pathway, sequentially articulating each step aloud. Verification was performed jointly with the assisting surgeon before proceeding further. This process was conceptually analogous to the verbal confirmation used during the Surgical Safety Checklist at the beginning and end of surgical procedures, providing a structured moment of shared verification focused on intraoperative quality rather than team logistics.

The time required to complete verbal recall of all nine CVMPO criteria was recorded intraoperatively as the CVMPO recall time during a predefined pause point. In bilateral procedures, verbal recall was performed separately for each side at two distinct intraoperative pauses; for analytical purposes, CVMPO recall time was calculated as the mean of the two recall intervals corresponding to each side. In contrast, the time required to complete corrective actions was determined postoperatively based on independent review of the operative video recordings.

### Corrective Actions and Workflow Integration

Following the verbal recall process, the operating surgeon verified whether any CVMPO elements required correction or optimization, and any identified deficiencies were promptly addressed intraoperatively. All identified corrective actions were addressed before mesh placement whenever technically feasible. Mesh placement was performed only after verification that all CVMPO criteria had been reviewed and fulfilled to the best technically achievable extent. At the conclusion of the procedure, final desufflation of the operative field was performed in accordance with established safety principles.

### Video-Based Verification

All procedures were recorded in full. Independent *post hoc* video review was performed to verify objective adherence to all CVMPO criteria. Video recordings were reviewed by two independent reviewers who were not involved in the respective procedures. Discrepancies in assessment were resolved by consensus review. Formal inter-rater reliability analysis was not performed. Reviewers were blinded to surgeon identity, and recordings contained only the laparoscopic video feed without audio, preventing awareness of intraoperative verbal recall or team communication. In cases where corrective actions were reported, video analysis was used to confirm their appropriate implementation. Complete CVMPO adherence was defined as fulfillment of all nine criteria, as confirmed by video review.

### Outcome Measures

Primary outcome measures included:

1. Time required for complete recall of all nine CVMPO criteria using the V–M Pathway,
2. Rate of complete CVMPO adherence confirmed by independent video review.

Secondary outcome measures included frequency of corrective actions and concordance between surgeon self-assessment and video verification.

### Ethical Considerations

The study was conducted as a prospective observational study within an institutional quality-improvement initiative focused on intraoperative workflow optimization and was approved by the local ethics committee. All data were anonymized prior to analysis.

A step-by-step demonstration of the V–M Pathway applied during laparoscopic inguinal hernia repair is provided as Supplementary Video.

## Results

During the study period, 225 laparoscopic inguinal hernia repairs were performed. Fourteen procedures were excluded due to incomplete recording (n = 11) or technical constraints preventing video retrieval (n = 3). A total of 211 procedures were therefore included in the final analysis. Complete recall of all nine CVMPO criteria using the V–M Pathway was achieved in all procedures, with a median recall time of 58 s (IQR 52–64; range 41–92). Median operative time was 49 min (IQR 45–56; range 29–78) for unilateral procedures and 62 min (IQR 57.5–68; range 49–82) for bilateral procedures. Baseline operative characteristics and intraoperative outcomes are summarized in Table 1.

**TABLE 1:** Intraoperative recall, corrective actions, and CVMPO adherence.

Variable	Value
Number of procedures	211
Unilateral repairs, n (%)	180 (85.3%)
Bilateral repairs, n (%)	31 (14.7%)
Median operative time, unilateral procedures, min (IQR; range)	49 (45–56; range 29–78)
Median operative time, bilateral procedures, min (IQR; range)	62 (57.5–68; range 49–82)
Median CVMPO recall time, sec (IQR; range)	58 (52–64; range 41–92)
Procedures requiring ≥1 corrective action, n (%)	43 (20.4%)
Median correction time among procedures requiring corrective actions, sec (IQR; range)	46 (38–81; range 12–270)
Complete CVMPO adherence on video review, n (%)	205 (97.2%)
Mesh placed only after CVMPO confirmation, n (%)	211 (100%)
Final desufflation performed, n (%)	194 (91.9%)

CVMPO recall time was measured intraoperatively during a predefined pause before mesh placement. In bilateral procedures, recall time represents the mean of two separate recall intervals performed for each side. Correction time was determined postoperatively based on independent video review and is reported only for procedures in which corrective actions were required. Complete adherence was defined as fulfillment of all nine CVMPO criteria on video assessment.

Intraoperative recall prompted at least one corrective action in 20.4% (43/211) of procedures. Among procedures requiring corrective actions, the median time needed to complete all corrections was 46 s (IQR 38–81; range 12–270). All corrective actions were completed before mesh placement whenever technically feasible.

This corresponded to approximately 1.5%–2% of total operative time. In procedures requiring corrective actions, the combined time for CVMPO recall and corrections had a median duration of 104 s, accounting for approximately 3% of total operative time.

Independent video review confirmed complete adherence to all CVMPO criteria in 97.2% (205/211) of procedures. Mesh placement was performed only after CVMPO confirmation in all cases; final deliberate desufflation of the preperitoneal space was performed in 91.9% (194/211) of procedures.

**FIGURE 2:**
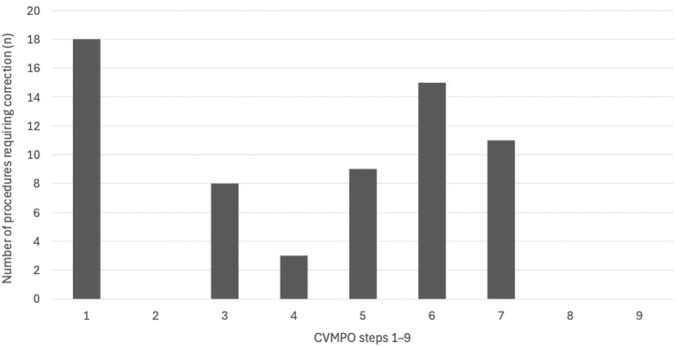
Distribution of corrective actions across CVMPO steps. Bars represent the number of procedures in which at least one corrective action was required for a given CVMPO step.

Corrective actions were not evenly distributed across CVMPO elements but clustered around a limited subset of criteria, as detailed in Table 2 and illustrated in Figure 2.

**TABLE 2:** Distribution of corrective actions by CVMPO element.

Step number	Step description	Value
1	Identify and dissect the pubic tubercle across the midline and the cooper ligament	18 (8.5%)
2	Rule out a direct hernia	0 (0%)
3	Dissect at least 2 cm between cooper ligament and the bladder	8 (3.8%)
4	Identify the femoral orifice and rule out a femoral hernia	3 (1.4%)
5	Dissect the indirect sac and peritoneum sufficiently to parietalize the cord’s elements	9 (4.3%)
6	Identify and reduce cord lipomas	15 (7.1%)
7	Dissect peritoneum lateral to the cord’s elements beyond the anterosuperior iliac spine	11 (5.2%)
8	Ensure adequate mesh coverage and fixation above the inter-ASIS line	0 (0%)
9	Place the mesh only after completion of steps 1–8 and verification of hemostasis	0 (0%)

Corrective actions were recorded intraoperatively following verbal CVMPO, recall using the V–M Pathway. The table reports the number and percentage of procedures in which a given CVMPO, element required correction. Multiple CVMPO, elements could be corrected within a single procedure.

## Discussion

This study shows that the V–M Pathway, a spatial framework integrated into laparoscopic inguinal hernia repair, can be incorporated into intraoperative workflow and was associated with high adherence to the CVMPO criteria while preserving operative workflow. By transforming a checklist into an anatomically mapped pathway, the framework is intended to address a recognized gap between knowledge of quality criteria and their consistent execution during real-time surgery. The present study design does not allow isolation of the individual contributions of the deliberate intraoperative pause, verbalization of criteria, assistant confirmation, or spatial mapping itself. The observed effects likely result from the combined use of a structured pause together with spatially organized recall. Future comparative studies are required to determine the relative contribution of each component.

The use of mnemonic checklists and structured cognitive frameworks is well established in other areas of surgery [10–13]. A prominent example is the Critical View of Safety in laparoscopic cholecystectomy, which provides a standardized visual checklist to prevent bile duct injury [14]. In laparoscopic surgery, structured checklists have been introduced as cognitive aids to support intraoperative workflow and minimize preventable technical problems, reflecting a broader shift toward standardized verification strategies beyond reliance on individual memory alone [15]. These examples illustrate that external cognitive structures can effectively support intraoperative decision-making. In contrast to these predominantly visual or conceptual checklists, the V–M Pathway integrates quality criteria directly into the laparoscopic anatomical field, providing spatial guidance tailored to minimally invasive inguinal hernia repair.

Incomplete fulfillment of individual CVMPO criteria may have clinically relevant consequences [16]. Insufficient mesh overlap in critical areas has been associated with an increased risk of hernia recurrence, whereas suboptimal mesh positioning or fixation may contribute to postoperative pain [1–4]. Additionally, failure to identify and address occult findings, such as lipomas or femoral hernias, may result in persistent symptoms or early recurrence [1–4, 16].

Analysis of the distribution of corrective actions suggests that not all CVMPO steps carry equivalent clinical implications when omitted. The V–M Pathway most frequently prompted verification of the presence of a cord lipoma, a step that may otherwise be overlooked, particularly in the absence of overt hernia anatomy. In contrast, corrective actions related to extended medial or lateral dissection, peritonealization of the triangle of doom, or adequate inferolateral dissection to accommodate the mesh primarily addressed technical prerequisites for optimal mesh placement. Omission of these steps would likely manifest as technical difficulty during mesh placement, potentially necessitating additional dissection with the mesh already present in the operative field. By comparison, failure to identify and reduce a cord lipoma carries a distinct risk of persistent symptoms or pseudo-recurrence [1–4]. These observations underscore the differential clinical relevance of individual CVMPO elements and highlight the value of structured verification in preventing omission of diagnostically significant findings.

From a practical perspective, corrective actions observed in this study may be broadly divided into minor technical optimizations facilitating safe and tension-free mesh deployment and corrections addressing findings with potential clinical consequences, such as insufficient medial dissection or failure to identify and reduce cord lipomas. The V–M Pathway appears to support systematic verification of both types of elements, helping prevent omission of clinically relevant findings while simultaneously improving technical conditions for mesh placement.

In a limited number of procedures, incomplete formal fulfillment of selected CVMPO criteria reflected deliberate surgical judgment rather than omission. These situations primarily involved patients with a history of robotic-assisted radical prostatectomy, where dense adhesions raised concern for bladder injury. In one case, the bladder was adherent to the pubic bone, and in another to the iliac vessels, rendering further dissection potentially hazardous. In the remaining cases, challenges occurred during repair of the contralateral side in patients with a history of prior inguinal hernia repair, where previously implanted mesh was found densely adherent to the bladder. In all such scenarios, the V–M Pathway fulfilled its intended role by explicitly prompting structured consideration of the relevant CVMPO elements, after which the surgeon consciously elected to limit further dissection to avoid undue risk. This underscores that the purpose of CVMPO verification is to support informed intraoperative decision-making rather than to mandate rigid technical compliance. Even when corrective actions were required, the time devoted to CVMPO verification and optimization represented only a small fraction of the overall procedure duration, indicating that integration of the pathway does not substantially disrupt operative workflow.

Notably, no corrective actions were required for several CVMPO steps, suggesting that vulnerability is confined to a limited subset of anatomical checkpoints rather than reflecting global procedural inconsistency [1, 2]. The absence of corrective actions related to mesh fixation reflects a deliberate institutional preference for limited or no fixation, with fixation applied only in selected cases and never below the inter-ASIS line, in accordance with CVMPO principles. Similarly, no corrective actions were recorded for the step requiring exclusion of a direct hernia, which is typically achieved as an inherent part of standard preperitoneal dissection.

An additional advantage of the V–M Pathway is its simplicity. The spatial structure is intuitive, easy to memorize, and readily associated with the laparoscopic view, allowing surgeons to apply it immediately after initial exposure without the need for formal training or repeated rehearsal. This low cognitive entry threshold may facilitate rapid adoption in routine clinical practice [17].

Importantly, corrective actions prompted by the framework were also observed in procedures perceived as technically straightforward and associated with low subjective fatigue, indicating that vulnerability of CVMPO elements is not limited to complex or demanding cases.

### Strengths and Limitations

Key strengths of this study include its pragmatic intraoperative implementation, detailed workflow description, and dual verification approach combining surgeon-reported behavior with independent video-based assessment. By focusing on structured intraoperative verification embedded directly within the operative field, the V–M Pathway addresses an underexplored aspect of surgical quality criteria that is directly translatable to routine clinical practice.

A key limitation of this study is the absence of a comparator or baseline cohort, which prevents assessment of whether adherence achieved with the V–M Pathway differs from standard practice in the absence of this intervention. Although the study was conducted in a standardized high-volume environment, the simplicity and spatial nature of the V–M Pathway may facilitate its application in less standardized settings or among lower-volume surgeons by providing structured intraoperative guidance. However, adoption and perceived usefulness may vary depending on surgeon experience and institutional workflow, and surgeons who have already internalized their own verification strategies may perceive less incremental benefit from adopting an additional cognitive framework. Results may partly reflect local institutional organization and operative culture, which may limit generalizability to centers with different surgical volumes or operative practices.

The primary aim of this study was to evaluate intraoperative usability and behavioral impact rather than comparative effectiveness, and the findings should therefore be interpreted as demonstrating feasibility and workflow integration. While this study evaluated the pathway in TAPP repair, the conceptual framework may also be adaptable to other minimally invasive approaches, such as TEP repair, although this requires dedicated evaluation.

Surgeons were aware that procedures were recorded and that adherence was being assessed, which may have introduced performance bias (Hawthorne effect) and contributed to higher adherence rates. Routine recording of laparoscopic procedures had already been established in the institution for several years prior to study initiation, and surgeons were therefore accustomed to operating under continuous recording conditions. This familiarity may reduce the magnitude of behavioral modification associated with observation over time; however, performance bias related specifically to study-related compliance assessment cannot be fully excluded. Surgeon self-assessment may also introduce reporting bias; however, this limitation was mitigated by independent *post hoc* video verification confirming objective adherence to CVMPO criteria.

The high adherence rates observed may partially reflect existing institutional expertise and standardized operative workflow, introducing a potential ceiling effect that may limit measurable incremental benefit in similarly high-volume or highly standardized centers. In addition, procedures were performed by surgeons at different stages of training, including residents in the early phases of independent laparoscopic practice. In such settings, structured spatial guidance may help standardize verification of key operative steps and reduce the risk of omission of individual CVMPO elements despite overall institutional experience.

The analysis was primarily descriptive and did not explore predictors of corrective actions or variability across surgeons or case complexity, which may be addressed in future studies. The study did not formally assess the learning curve associated with adoption of the V–M Pathway, and long-term sustainability of pathway use beyond monitored study conditions was also not evaluated. It therefore remains unclear how quickly surgeons unfamiliar with the framework can internalize and consistently apply it in routine practice, and whether adherence to structured cognitive aids is maintained once external monitoring is reduced. Future studies should examine long-term adoption patterns and user acceptance in routine clinical settings. Finally, future investigations should evaluate the long-term clinical impact of improved intraoperative verification strategies.

### Conclusions

The V–M Pathway provides a simple spatial cognitive aid for intraoperative verification of CVMPO criteria during laparoscopic inguinal hernia repair. Its integration as a structured intraoperative pause before mesh placement supported rapid recall, prompted timely corrective actions, and was associated with high video-verified adherence without disrupting operative workflow.

By providing a structured spatial framework for intraoperative verification rather than relying solely on technical execution, the V–M Pathway represents a potentially scalable approach to supporting procedural consistency and adherence to quality criteria in minimally invasive inguinal hernia surgery. However, in the absence of comparator data, the findings should be interpreted as demonstrating feasibility and workflow integration rather than definitive improvement over standard practice. Further studies are warranted to evaluate its impact across broader practice settings and on long-term clinical outcomes.

## Data Availability

The raw data supporting the conclusions of this article will be made available by the authors, without undue reservation.
